# Editorial on the Special Issue “Modification of Gels in Creating New Food Products”

**DOI:** 10.3390/gels11110903

**Published:** 2025-11-11

**Authors:** Anna Florowska, Tomasz Florowski, Osvaldo H. Campanella

**Affiliations:** 1 Department of Food Technology and Assessment, Institute of Food Sciences, Warsaw University of Life Sciences-SGGW, 02-787 Warsaw, Poland; tomasz_florowski@sggw.edu.pl; 2 Department of Food Science and Technology, The Ohio State University, Columbus, OH 43210, USA

The strategic modification of gel systems constitutes a fundamental direction in contemporary food science, enabling the design of products with enhanced functionality, nutritional value, and consumer acceptability. Gels play a critical role not only in determining the rheological and sensory characteristics of food matrices but also in supporting the structural integrity, processability, and stability of diverse formulations. In light of increasing consumer interest in plant-based [1], fiber-rich [2], and fat-reduced products [3], research efforts have focused on tailoring gel systems by incorporating biopolymers, applying advanced processing technologies, and modulating physicochemical interactions within composite matrices.

This Special Issue concerns the modification of gels in creating new food products. All published articles focus on the deliberate modification of gel structures to achieve specific improvements in food quality and functionality. The studies cover a range of food systems, including cereal-based products (contribution 1), protein gels (contributions 2, 3), oleogels (contributions 4, 5), marine origin ingredients as elements of gels (contributions 6–8), functional products (contributions 9, 10), and edible hydrogel films (contribution 11). Despite the diversity of applications, these contributions converge on a shared objective: the controlled manipulation of gel systems to yield optimized structural and functional outcomes in line with current demands for technological efficiency, sensory performance, and nutritional relevance.

A representative example of this approach is provided by the work on early indica rice, which is a case of gel modification in the improvement of the cooking quality. Early indica rice is a widely grown, but texturally suboptimal cereal. In the research of Wang et al. (contribution 1), the incorporation of polydextrose (ST and XG types with different moisture content) at concentrations from 3 to 10% to the cooking milled rice polished was shown to significantly improve cooking quality and reduce the hard, chewy texture characteristic of this rice variety. Both forms of polydextrose reduced cooking time, increased gruel solids loss, and decreased key textural parameters, including hardness, adhesiveness, and gumminess. Thermo-rheological analyses revealed reductions in viscosity and gelatinization enthalpy, indicating a disruption of starch retrogradation mechanisms. Microstructural imaging confirmed enhanced water penetration and internal loosening of the starch matrix, leading to more uniform swelling and softer kernels. Notably, XG polydextrose type was more effective in reducing retrogradation, while ST type strongly suppressed gelatinization enthalpy. These results suggest that polydextrose can be effectively applied to improve the sensory quality and preparation efficiency of early indica rice, offering a functional solution for enhancing staple foods in high-volume production contexts (contribution 1).

Another thematic area covered in this Special Issue is the design and modification of protein-based gels, which play a pivotal role in developing foods with tailored texture, stability, and functionality. Two studies, described by Florowska et al. (contribution 2) and Teng and Campanella (contribution 3), demonstrate how different gelling strategies can be harnessed to produce protein-containing structures. The research by Florowska et al. (contribution 2) investigates the use of high hydrostatic pressure (HHP) as a non-thermal technology to induce gelation in systems composed of inulin and soy protein isolate (SPI). By systematically varying protein concentration (3% and 6%) and processing parameters (150–500 MPa; 5–20 min), the study identifies 300 MPa for 5 min as the most effective condition for producing hydrogels with desirable mechanical attributes, particularly in formulations containing 3% SPI. Under these conditions, the gels exhibited greater firmness, adhesiveness, yield stress, and reduced spreadability, resulting in compact and cohesive structures compared to those obtained at 150 MPa. Importantly, the process enabled gel formation at ambient temperature, preserving thermosensitive compounds and highlighting the potential of HHP for creating functional matrices with controlled textural properties and delayed release characteristics. The article of Teng and Campanella (contribution 3) addresses a different application of protein-based gel systems: the creation of fully plant-based fat analogs for meat alternatives. A two-step gelation method combined sodium alginate, pea protein isolate, and soybean oil to produce gels containing up to 70% oil, with oil droplets stably encapsulated within a protein-stabilized alginate network. Calcium-induced gelation yielded a light-yellow, visually fat-like material with viscoelastic properties and thermal behavior closely resembling animal fat. The structural confinement of oil within the gel delayed its release during heating, effectively imitating the slow-rendering characteristics of beef fat. Analytical techniques (FTIR, DSC) confirmed the absence of chemical modification during gelation, ensuring product safety and stability. This approach offers a scalable, clean-label alternative to saturated fats in plant-based meat formulations, combining sensory quality with improved nutritional profiles.

Beyond water-based gel systems, another important research direction in food structuring focuses on oleogels—semi-solid systems in which liquid oils are immobilized within a three-dimensional network without using saturated or hydrogenated fats. Such systems are attracting increasing attention for their potential to replace conventional animal-derived fats while maintaining desirable texture, stability, and sensory characteristics. Two contributions in this Special Issue— Articles Hong et al. (contribution 4) and Harasym and Banaś (contribution 5) —highlight both the practical development and the conceptual framework for oleogelation, with particular emphasis on the use of lecithin and natural hydrocolloids. Hong et al. (contribution 4) present a formulation of turmeric extract–enriched oleogels (TE-OGs) structured via an emulsion-template method using xanthan gum and soy lecithin. Optimized concentrations of 0.32% xanthan gum and 1.2% lecithin yielded a gel with exceptionally high oil-binding capacity (>99.9%), solid-like viscoelastic properties, and notable thermal stability. The oleogel functioned as a carrier for bioactive curcumin. It showed limited release under gastric conditions and sustained release in simulated intestinal fluid—up to 70%—demonstrating its potential for targeted delivery of lipophilic compounds. Application trials in pound cake, where TE-OGs partially replaced butter, resulted in enhanced crumb softness and porosity without compromising sensory acceptance. The article by Harasym and Banaś (contribution 5) reviews lecithin’s roles in oleogelation, categorizing its contributions into structural, functional, and technological domains. As an oleogelator and emulsifier, lecithin stabilizes dispersed oil phases, promotes network formation, and enhances oil-binding capacity. It influences crystallization behavior, modulates gel texture, and improves thermal stability and viscoelasticity. Lecithin also exhibits synergistic effects with other oleogelators, such as waxes and hydrocolloids, by reinforcing hydrogen bonding and facilitating the self-assembly of ordered structures within the gel matrix. From a functional standpoint, lecithin improves the bioavailability of lipophilic nutrients through micelle and liposome formation and can contribute to oxidative stability, extending product shelf life. Its multifunctionality aligns well with clean-label trends, reducing the need for multiple additives in a single formulation.

A further thematic focus in this Special Issue is the utilization of marine-derived ingredients in gel systems, reflecting both their functional versatility and alignment with sustainability goals. Researchers are developing gels with tailored mechanical properties, bioactive potential, and clean-label appeal by harnessing proteins and polysaccharides from seafood byproducts and marine biomass. Articles Ramírez-Campas et al. (contribution 6), Charoenphun et al. (contribution 7), and Abbas et al. (contribution 8) illustrate complementary strategies in this domain, spanning composite hydrogel design, plant-based gelling agents from seaweed, and the reformulation of traditional seafood products. One of the studies examines composite, antioxidant hydrogels formed from chitosan and gelatin extracted from squid-processing byproducts. Varying the biopolymer ratio in the hydrogels enabled fine-tuning of mechanical performance: increasing the gelatin fraction reduced hardness and chewiness, while enhancing springiness, resilience, and antioxidant activity. Structural compatibility between chitosan and gelatin, confirmed via spectroscopic and morphological analyses, facilitated the formation of porous and stable networks. Beyond their structural role, the antioxidant capacity of gelatin-rich formulations positions these hydrogels as potential carriers for bioactive compounds. Such systems hold promise for applications where elasticity and a soft gel texture are desired, including restructured seafood products, functional jellies, and edible coatings, while supporting marine byproduct valorization (contribution 6). Another study focuses on *Gracilaria fisheri* (GF), a red seaweed rich in gelling polysaccharides, as a natural structuring agent in a strawberry-based drinking jelly. Using GF at concentrations of 0.2–1.0%, the study identified 0.8% as optimal, yielding a gel with an optimal color balance in lightness and redness, visually appealing appearance, and stable matrix formation while retaining drinkability. Rheological measurements showed that increasing GF levels led to higher viscosity and gel strength, but at the highest concentration (1.0%), the gels became too thick, reducing drinkability. Antioxidant content decreased slightly at higher concentrations, but volatile analysis indicated that the optimal GF level (0.8%) supported aroma retention and gradual release, enhancing fruity and floral sensory notes. The findings highlight GF’s capacity to deliver structural integrity and sensory quality in low-viscosity gel systems, underscoring its potential in clean-label and plant-based formulations (contribution 7). The research by Abbas et al. (contribution 8) addresses the reformulation of low-salt Silver Carp (*Hypophthalmichthys molitrix*) surimi by incorporating psyllium husk powder (PHP) as a functional structuring agent. At concentrations of 0.1–0.3%, PHP significantly improved gel strength, water-holding capacity, and network stability through enhanced protein–polysaccharide interactions. The additive promoted disulfide bonding and hydrophobic interactions among myofibrillar proteins, producing firmer, more elastic, and more cohesive gels—outcomes supported by favorable sensory evaluations. This approach demonstrates how plant-derived fibers can synergistically interact with marine proteins to offset the technological effects of reduced salt, enabling the production of healthier surimi products without compromising consumer acceptance (contribution 8).

An additional direction highlighted in this Special Issue concerns the use of gel matrices as vehicles for functional compounds, with particular emphasis on prebiotics and probiotics. Integrating bioactive ingredients into structured food systems makes enhancing nutritional value and tailoring texture, stability, and sensory appeal possible. Two contributions, i.e., Vigil-Cuate et al. (contribution 9) and Li et al. (contribution 10), provide complementary perspectives on this theme, focusing on synbiotic confectionery and fermented dairy gels. Vigil-Cuate et al. (contribution 9) study explores the development of gelatin-based gummies enriched with agavins and agave syrup, alongside alginate-encapsulated *Saccharomyces boulardii*. Replacing conventional sweeteners (sucrose) with agavins modified the gel network, increasing water activity and cohesiveness while reducing hardness, resulting in a soft, elastic, and easy-to-swallow confection. These structural effects were linked to the hygroscopic nature of agavins, which promoted water retention. Crucially, the incorporation of alginate microcapsules did not compromise the gummy structure but provided a supportive environment for the probiotics, maintaining their metabolic activity over 24 days in a viable but non-culturable state. This formulation demonstrates the feasibility of synbiotic gummies with appealing texture and extended probiotic viability, offering substantial potential for functional confectionery markets. In the study of Li et al. (contribution 10), prebiotic compounds were introduced into dairy gel systems during yogurt fermentation to evaluate their impact on texture and stability. Comparative analysis of five prebiotics—fructooligosaccharide (FOS), galactooligosaccharide (GOS), inulin (INU), polydextrose (PDX), and xylooligosaccharide (XOS)—revealed that INU, PDX, and XOS significantly strengthened the protein gel network. These compounds improved firmness, cohesiveness, and water retention, while reducing whey separation, effects attributed to their higher molecular weight and stronger interactions with casein micelles. By contrast, FOS and GOS primarily enhanced viscosity without markedly reinforcing the gel matrix. Such results highlight the potential of selected prebiotics to modulate not only nutritional functionality but also key rheological and sensory attributes, enabling the production of yogurts with improved consistency, reduced syneresis, and enhanced consumer appeal. These two studies illustrate the capacity of gel matrices to serve as multifunctional platforms, providing structural integrity while acting as vehicles for the incorporation and stabilization of bioactive compounds. Whether applied in confectionery or fermented dairy, the strategic integration of pre- and probiotics into gels demonstrates how nutritional enhancement can be achieved alongside desirable sensory properties, supporting the expanding market for health-oriented functional foods.

Beyond their role in structuring foods and delivering bioactive compounds, hydrogels are also being explored as sustainable alternatives to conventional packaging materials. In response to environmental concerns associated with plastic waste, hydrogel-based membranes derived from renewable or waste resources offer a promising strategy for developing edible and biodegradable packaging solutions. A study within this Special Issue reported on the fabrication of hydrogel films based on casein proteins recovered from expired dairy products, formulated with chitosan and glycerol as a plasticizer (contribution 11). Incorporating casein increased the membranes’ elasticity, while glycerol enhanced strain tolerance, thereby improving handling properties. The formulation containing equal proportions of chitosan and casein (Chi50Cas50) with glycerol provided an optimal balance of mechanical strength and flexibility. Importantly, these membranes exhibited zero oxygen permeability alongside a low glass transition temperature, indicating their suitability for use in protecting foods under both refrigerated and ambient conditions. The findings position chitosan–casein hydrogel membranes as viable alternatives to petroleum-derived packaging, combining functional performance with a reduced environmental footprint. The valorization of expired dairy as a raw material further strengthens the sustainability credentials of this approach. Such materials hold considerable potential as edible or biodegradable barrier layers for fresh foods, where the exclusion of oxygen and maintenance of mechanical pliability are critical to quality preservation (contribution 11).

The articles collected in this Special Issue, Modification of Gels in Creating New Food Products, collectively demonstrate the breadth of approaches by which gel systems can be engineered to meet contemporary challenges in food design. Across diverse studies, a unifying theme emerges: gels provide versatile structural platforms tailored to deliver targeted textural, functional, and nutritional properties while aligning with sustainability goals.

The studies presented in this Special Issue underscore the central role of gels in the next generation of food innovation. Gel systems provide a flexible technological platform that bridges consumer expectations with industrial needs by enabling precise control over texture, stability, nutrient delivery, and environmental performance. Future research in this field will likely expand on these foundations, integrating materials science, biotechnology, and sustainability advances to design foods and food-related materials with increasingly sophisticated and purposeful properties.
